# Thermal Ablation of Breast Cancer Liver Metastases Is Associated with Durable Local Control and Chemotherapy-Free Intervals in Selected Patients

**DOI:** 10.3390/cancers18121997

**Published:** 2026-06-19

**Authors:** Niaz Ahmed, Alicia Okines, Sophie McGrath, Marina Parton, Emma Kipps, Nicholas Turner, Edward Johnston, Stephen Johnston, Nicos Fotiadis

**Affiliations:** The Royal Marsden Hospital, Fulham Road, London SW3 6JJ, UK; niaz.ahmed1@nhs.net (N.A.); alicia.okines@rmh.nhs.uk (A.O.); sophie.mcgrath@rmh.nhs.uk (S.M.); marina.parton@rmh.nhs.uk (M.P.); emma.kipps@rmh.nhs.uk (E.K.); nicholas.turner@rmh.nhs.uk (N.T.); ed.johnston@rmh.nhs.uk (E.J.);

**Keywords:** breast cancer, liver metastases, ablation, microwave ablation, radiofrequency ablation, oligometastatic, oligoprogression, oligopersistence

## Abstract

**Background/Objectives**: In selected patients with oligometastatic breast cancer liver metastases (BCLM), liver-directed therapies may provide durable local control and may delay escalation of systemic therapy. This study reports a single-center experience of percutaneous thermal ablation (radiofrequency ablation [RFA] or microwave ablation [MWA]) for BCLM, including conventional oncologic outcomes and therapy-based endpoints. **Methods**: This retrospective cohort included consecutive patients treated with percutaneous ablation for BCLM following multidisciplinary team approval between 2005 and 2025. Outcomes were defined according to the Society of Interventional Oncology (SIO) and DATECAN consensus terminology. Lesion-level outcomes included primary/secondary technique efficacy and local tumor progression-free survival (LTPFS). Patient-level outcomes included progression-free survival (PFS), overall survival (OS), time to change in systemic therapy (TTCST) and chemotherapy-free survival (CFS). Kaplan–Meier and Cox regression analyses were performed. **Results**: Forty-six patients underwent 58 ablation sessions treating 80 metastases (median tumor size 19 mm, interquartile range [IQR] 13–27 mm). Primary and secondary technique efficacy were 95% (76/80) and 99% (79/80), respectively. Major complications occurred in 2/58 sessions (3%). Local tumor progression occurred in 16/79 tumors (20%) after a median follow-up of 28 months; LTPFS rates at 1, 3 and 5 years were 84%, 75% and 75%, respectively. Median OS was 44 months (1-, 3- and 5-year OS 94%, 58%, and 40%), and median PFS was 8.3 months. Median TTCST was 13 months, and median CFS was 16.4 months. Triple-negative disease was associated with worse LTPFS and shorter CFS. Oligopersistent disease was associated with improved PFS compared with oligoprogression. **Conclusions**: In this selected cohort, percutaneous thermal ablation for BCLM achieved high technique efficacy, durable local control and low major complication rates. Therapy-based endpoints suggest a clinically meaningful interval without systemic therapy escalation in appropriately selected patients, although comparative studies are needed to quantify the incremental benefit.

## Simple Summary

Some people with breast cancer develop a small number of cancer deposits in the liver. In carefully selected patients, these deposits can sometimes be treated by thermal ablation, a procedure that uses image-guided needles to heat and destroy tumors. This study looked back at 46 patients treated at one center over 20 years to see how well this approach worked, how safe it was, and whether it helped delay changes in cancer drug treatment. The treatment was technically successful in almost all treated tumors, with a low rate of major complications. Local tumor control was maintained in many patients for several years. On average, patients went about 13 months before needing a change in cancer drug treatment. These findings suggest that liver ablation may be a useful option for selected patients, although comparative studies are needed.

## 1. Introduction

Breast cancer is one of the leading causes of cancer mortality globally [1]. The development of breast cancer liver metastases (BCLM) occurs in 30–50% of metastatic breast cancer patients [2,3], and historically portends a poor prognosis with a five-year survival of 9–14% in patients receiving systemic therapy alone [3,4]. With modern treatment, median survival times for patients with advanced breast cancer have improved substantially for all subtypes, but liver metastases remain a poor prognostic feature [5].

Locoregional therapies for selected patients with oligometastatic BCLM have been associated with a progression-free survival advantage over systemic therapies alone [6]. Surgery, percutaneous ablation and stereotactic body radiotherapy (SBRT) are all commonly used treatment modalities [7,8,9,10,11,12]. In selected patients with oligometastatic BCLM, local therapies such as hepatic resection, percutaneous ablation and SBRT have each demonstrated high local control rates and encouraging long-term survival when added to systemic therapy. Retrospective series and meta-analyses suggest that resection generally achieves the longest OS, while ablation and SBRT can offer more comparable disease control in patients who are not surgical candidates, albeit with substantial selection bias and no head-to-head randomized data [13,14,15]. Furthermore, several of these studies have sought to identify prognostic factors such as tumor size, presence of extra-hepatic disease and molecular subtype.

This study presents a single-center experience of percutaneous ablation for BCLM, assessing conventional oncologic outcomes (local tumor control, progression-free survival and overall survival) alongside therapy-based endpoints, including time to change in systemic therapy (TTCST) and chemotherapy-free survival (CFS), which are increasingly recognized as clinically meaningful measures of treatment burden and quality of life [16,17,18,19,20]. In addition, we explore whether treatment intent for oligoprogression or oligopersistence, and breast cancer molecular subtype (hormone receptor-positive, HER2-positive and triple-negative disease) are associated with these outcomes in a selected oligometastatic population.

## 2. Materials and Methods

### 2.1. Patient Selection

Between 2005 and 2025, 46 consecutive patients with BCLMs were treated with percutaneous ablation (PA) using either radiofrequency or microwave energy.

Inclusion criteria were patients with an age ≥ 18, confirmed diagnosis of breast cancer liver metastasis on imaging and/or biopsy. The decision to pursue PA was made through multidisciplinary tumor board discussions that included input from a team of experienced medical oncologists, radiation oncologists, surgeons, diagnostic radiologists and interventional radiologists. Exclusion criteria included uncorrectable coagulopathy, poor performance status, uncontrolled extra-hepatic disease and inability to consent.

Treatment intent was divided into local consolidation of either oligoprogressive or oligopersistent states. Oligoprogression was defined as localized progression of a few metastatic lesions in the setting of otherwise controlled disease on systemic therapy, whereas oligopersistence was defined as the presence of a limited number of metastatic lesions that remained stable or showed only partial response on imaging after systemic therapy [21,22,23].

### 2.2. Procedure

Of the 80 metastases treated, 32 (40%) were treated with radiofrequency ablation (RFA) and 48 (60%) with microwave ablation (MWA). Reflecting technological evolution over the study period, MWA was used preferentially in the later years of the cohort and for larger and/or perivascular tumors. Similarly, the use of a stereotactic guidance system and image fusion was used in later years, reflecting the availability of improved technologies. A minimal ablation margin of >5 mm beyond the visible tumor was targeted where anatomically feasible. All procedures were performed under general anesthesia by a radiologist. Either microwave or radiofrequency applicators were inserted into the liver using CT or combined US and CT guidance. Specific generator and applicator details were not consistently available across the full 20-year study period and were therefore not analyzed.

### 2.3. Follow-Up

Follow-up consisted of cross-sectional imaging within 6–12 weeks followed by imaging at 3- to 6-month intervals. This was predominantly with contrast-enhanced CT and/or contrast-enhanced MRI.

### 2.4. Statistical Analysis

For each patient and procedure, the following data were collected: patient demographics (age, sex), date of breast cancer diagnosis, breast cancer molecular subtype and BRCA status at the time of diagnosis, procedural details, hospital duration, imaging follow-up and outcomes, including complications. A history of oncologic therapies and changes to systemic treatment were also recorded.

The Kaplan–Meier method was used to calculate local tumor progression-free survival (LTPFS), progression-free survival (PFS), overall survival (OS), time to change in systemic therapy (TTCST) and chemotherapy-free survival (CFS). Primary and secondary technique efficacy, complications and time-to-event oncologic outcomes were analyzed. For time-to-event analyses, patients or lesions without the event of interest were censored at the date of last available clinical or imaging follow-up, respectively. Cox regression analysis was used to explore independent predictors of events. All Cox regression analyses were univariable. The number of events for each endpoint was: 16 local tumor progression events (LTPFS), 29 deaths (OS), 39 progression events (PFS), 34 changes in systemic therapy (TTCST) and 26 transitions to cytotoxic chemotherapy (CFS). Given that the molecular subtype is a three-level variable and principal subgroups of interest are small, the events per variable available for limiting endpoints (particularly LTPFS) fall below the conventional threshold required for stable multivariable estimation. To avoid overfitting and unstable, uninterpretable hazard ratios, multivariable modeling was therefore not undertaken, and all subgroup analyses are reported as univariable, exploratory and hypothesis-generating.

Complications were graded according to the Common Terminology Criteria for Adverse Events (CTCAE) standards [24]. Outcome and survival terms were defined according to the Society of Interventional Oncology (SIO) and the Definition for the Assessment of Time-to-Event End Points (DATECAN) initiative consensus guidelines [25]. Time to change in systemic chemotherapy (TTCST) was defined as the interval from first ablation to the first documented change in the systemic regimen for any reason. Chemotherapy-free survival (CFS) was defined as the interval from the first ablation to the initiation of cytotoxic chemotherapy or death from any cause, whichever occurred first; patients receiving endocrine or HER2-targeted therapy without cytotoxic chemotherapy were considered chemotherapy-free.

Survival analysis was performed using R v4.5.2 (R Foundation for Statistical Computing, Vienna, Austria).

## 3. Results

### 3.1. Patient Characteristics

Eighty breast cancer liver metastases (median size, 19 mm [interquartile range, 13–27 mm]) were treated in 46 patients across 58 sessions. Table 1 demonstrates the baseline characteristics of the study group.

The majority of patients (57%) were hormone receptor positive and HER2 negative (HR+). Thirteen patients (28%) were HER2 positive with either positive or negative hormone receptors (HER2+). The remainder were triple negative (TN). As a corollary, most patients were on some form of targeted therapy at the time of ablation, with very few on cytotoxic chemotherapy.

### 3.2. Complications

The major complication rate was 3% (2/58). One patient developed a symptomatic pneumothorax, which required prolonged observation (CTCAE grade 3). Another patient developed a hepatic vein thrombus, which, in the context of a prior left-sided hepatectomy, resulted in acute liver failure (CTCAE grade 4). This required catheter-directed thrombectomy and thrombolysis to good effect. There was one minor complication (CTCAE grade 1)—a biloma which only required imaging follow-up and spontaneously regressed.

The mean length of hospital stay was 1.5 days, ranging from 1 to 15 days.

### 3.3. Local Tumor Control

There were 4 cases of residual unablated tumor (RUT), yielding a primary technique efficacy of 95% (76/80). Three of these tumors were ablated again successfully, giving a secondary technique efficacy of 99% (79/80). The remaining tumor with RUT was not re-ablated and was excluded from the LTPFS analysis, leaving 79 tumors evaluable for local tumor progression.

**Figure 1:**
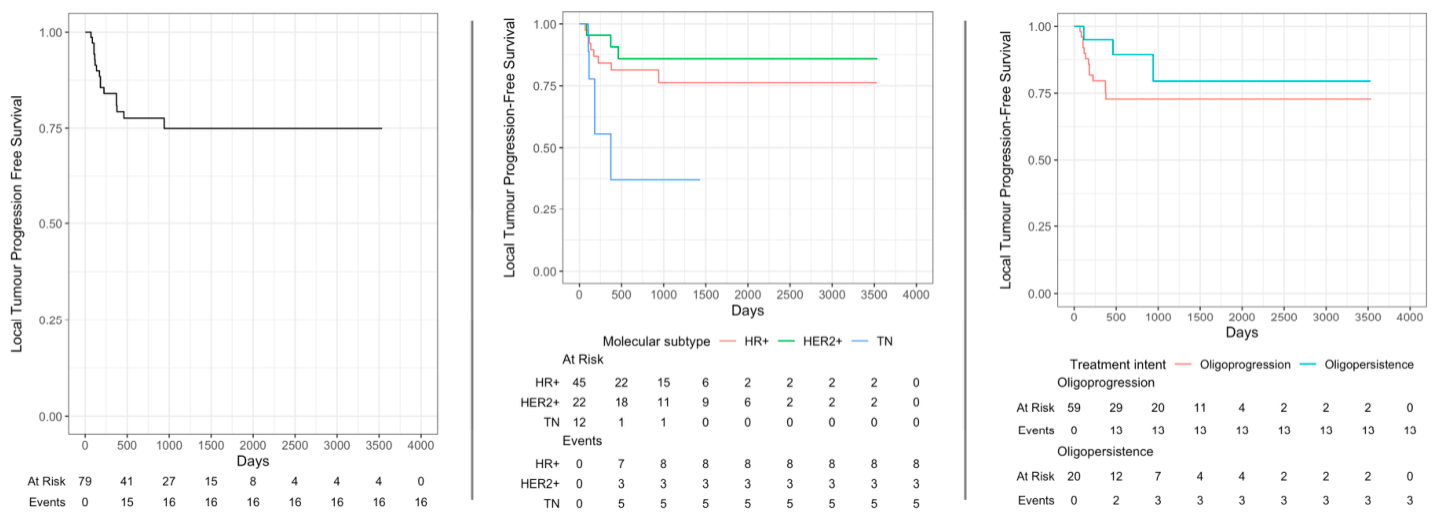
Overall LTPFS (**left**). LTPFS by molecular subtype (**middle**), where 1—HR+, 2—HER2+ and 3—TN. LTPFS by treatment intent (**right**), where 1—oligoprogression and 2—oligopersistence.

Local tumor progression occurred in 16 of 79 tumors (20%) after a median of 28 months. LTPFS rates at 1, 3 and 5 years from the date of first ablation were 84%, 75% and 75% (Figure 1).

In univariable analysis, molecular subtype was significantly associated with LTPFS (likelihood ratio test, *p* = 0.04). Whilst HER2+ did not show a statistically significant difference compared to HR+ in terms of LTPFS (HR 0.64, 95% CI 0.23–1.82, *p* = 0.40), TN metastases did have significantly worse LTPFS (HR 3.80, 95% CI 1.22–11.89, *p* = 0.02). Finally, there was a non-significant tendency towards improved LTPFS in patients treated for oligopersistence compared with oligoprogression (HR 0.5, 95% CI 0.14–1.76, *p* = 0.28).

In an exploratory analysis stratified by ablation modality, LTPFS did not differ significantly between RFA and MWA (HR 0.78, 95% CI 0.29–2.09, *p* = 0.63).

### 3.4. Overall and Progression-Free Survival

The median overall survival time was 44 months. OS rates at 1, 3 and 5 years from the date of the first ablation were 94%, 58% and 40%.

**Figure 2:**
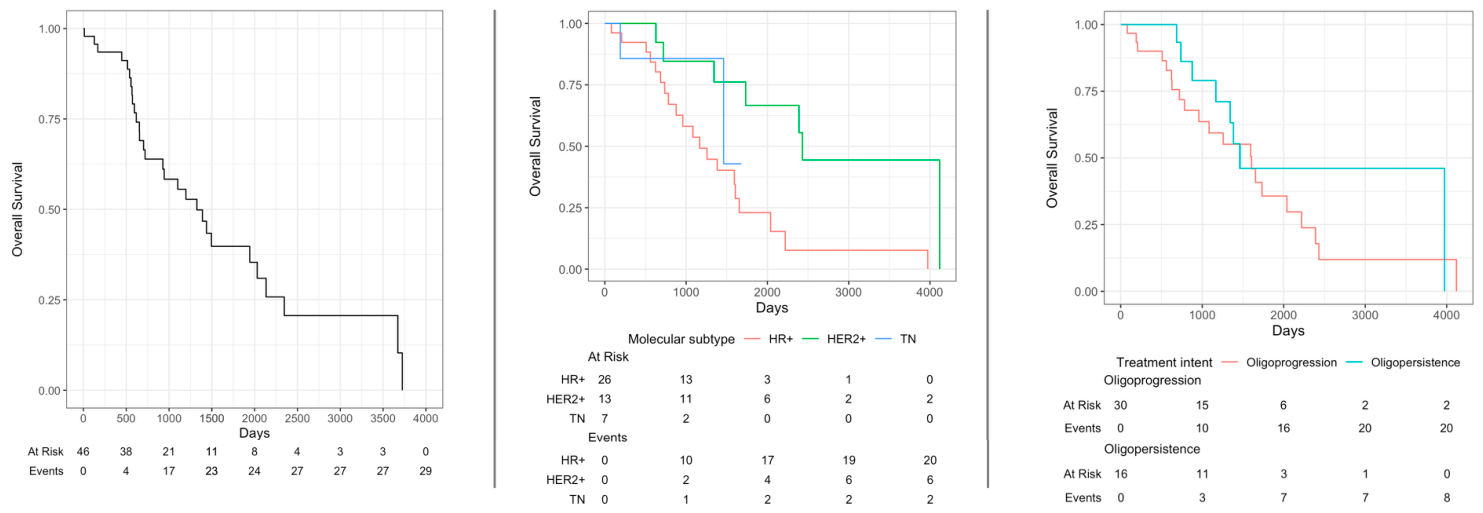
Overall OS (**left**). OS by molecular subtype (**middle**), where 1—HR+, 2—HER2+ and 3—TN. OS by treatment intent (**right**), where 1—oligoprogression and 2—oligopersistence.

In univariable analysis, compared with HR+ patients, HER2+ patients had significantly improved OS (HR 0.28, 95% CI 0.11–0.71, *p* = 0.008), whereas surprisingly, TN showed no clear difference. There was no significant difference in OS between oligoprogressive and oligopersistent patients (Figure 2).

The median progression-free survival time was 8.3 months from initial ablation. PFS rates at 1, 3 and 5 years from the date of the first ablation were 35%, 20% and 9%.

**Figure 3:**
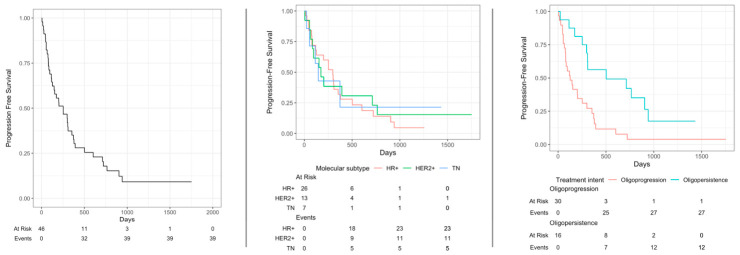
Overall PFS (**left**). PFS by molecular subtype (**middle**), where 1—HR+, 2—HER2+ and 3—TN. PFS by treatment intent (**right**), where 1—oligoprogression and 2—oligopersistence.

Univariable analysis suggests that patients treated for oligopersistence had a substantially lower hazard of progression compared with those treated for oligoprogression (HR 0.35, 95%, CI 0.17–0.71, *p* = 0.004). There was no significant difference in PFS between molecular subtypes (Figure 3).

### 3.5. Therapy-Based Outcomes

Median time to change in systemic therapy (TTCST) is 13 months. Three patients were excluded from this analysis because no clinical follow-up was available to ascertain the timing of therapy change.

**Figure 4:**
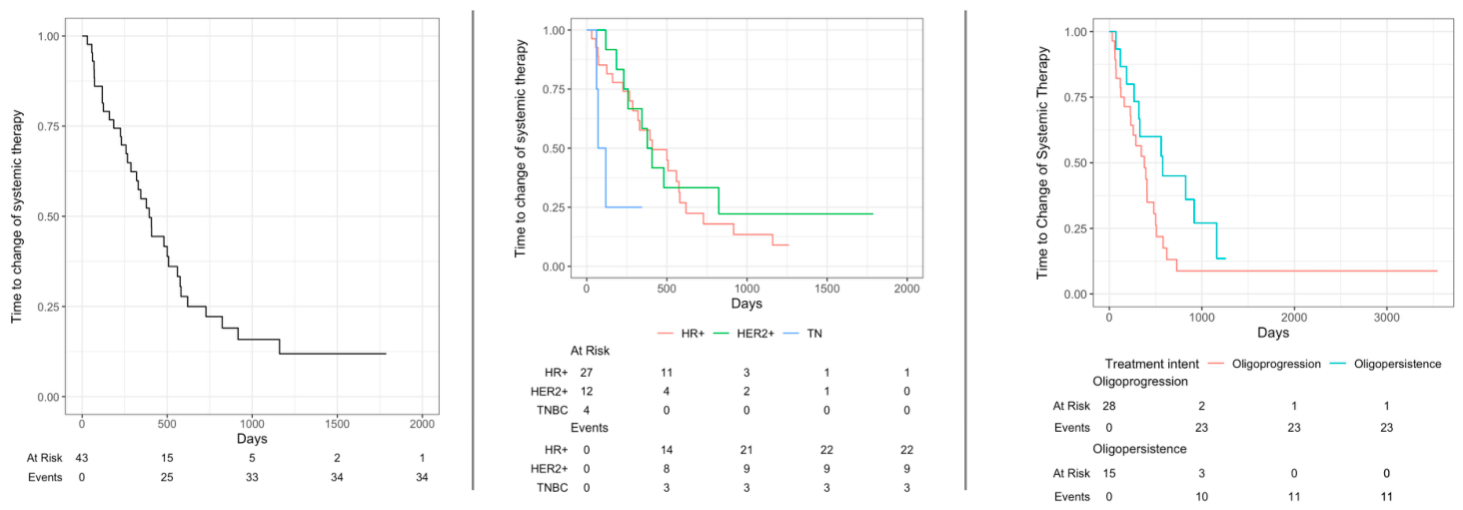
Overall TTCST (**left**). TTCST by molecular subtype (**middle**), where 1—HR+, 2—HER2+ and 3—TN. TTCST by treatment intent (**right**), where 1—oligoprogression and 2—oligopersistence.

There was a non-significant tendency to shorter TTCST in patients with TN (HR 3.36, 95% CI 0.94–11.98, *p* = 0.06). There was no significant difference in TTCST between oligoprogressive and oligopersistent patients (Figure 4).

The median chemotherapy-free survival was 16.4 months. Eight patients were excluded: five were already receiving cytotoxic chemotherapy at the time of ablation, and three had no clinical follow-up. The five patients excluded for baseline chemotherapy represented a more heavily pre-treated subgroup, so their exclusion may bias CFS estimates upward. In a sensitivity analysis, the five patients receiving cytotoxic chemotherapy at baseline were reincluded and coded as having experienced a CFS event at time zero. Median CFS changed modestly from 16.4 to 15.8 months, suggesting that the primary CFS estimate was robust to their exclusion.

**Figure 5:**
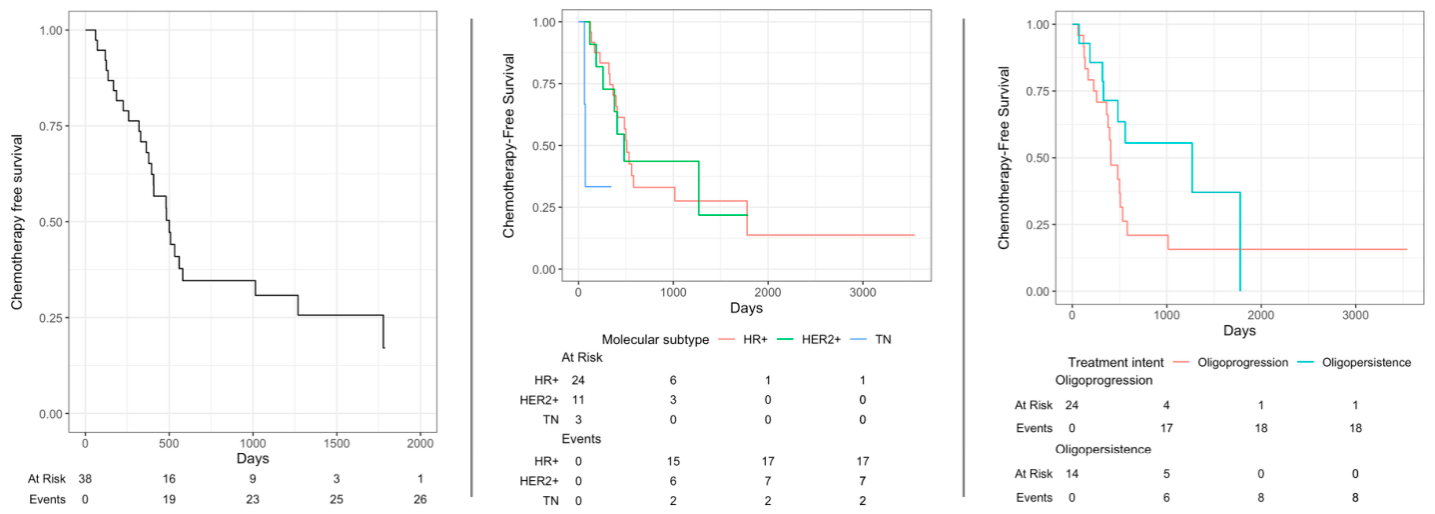
Overall CFS (**left**). CFS by molecular subtype (**middle**), where 1—HR+, 2—HER2+ and 3—TN. CFS by treatment intent (**right**), where 1—oligoprogression and 2—oligopersistence.

Patients with TN had a tendency to shorter CFS compared to HR+ patients (HR 5.64, 95% CI 1.17–27.09, *p* = 0.03). There was no significant difference for HER2+ patients or between oligoprogressive and oligopersistent states (Figure 5).

## 4. Discussion

In this single-center experience spanning two decades, percutaneous thermal ablation for breast cancer liver metastases (BCLM) achieved high technical success, durable local tumor control and low morbidity, while also demonstrating clinically meaningful systemic therapy-centered outcomes. Five-year local tumor progression-free survival reached 75% with a median overall survival of 44 months and a major complication rate of only 3%. Importantly, ablation was associated with a median delay of 13 months to systemic therapy change and a chemotherapy-free survival of 16.4 months, outcomes that are increasingly recognized as highly relevant to patients and oncologists alike.

Historically, the prognosis of BCLM treated with systemic therapy alone has been poor, with reported five-year survival rates below 15% in historic series [3,4]. Against this background, liver-directed therapies have substantially improved with long durations of disease control on first-line targeted therapies possible for both ER-positive [20] and HER-2 positive subtypes [26]. First-line therapies are often well-tolerated, with both patients and clinicians hesitant to change treatment for oligoprogression if local control can be regained.

Surgical series consistently report the longest survival outcomes, with median overall survival approaching 40–50 months in highly selected cohorts [4,27]. However, only a minority of patients are suitable for hepatic resection due to tumor distribution, comorbidities or prior liver surgery. Percutaneous ablation therefore occupies an important niche as a minimally invasive, repeatable, parenchyma-sparing alternative.

The oncologic outcomes observed in the present study compare favorably with existing ablation series in BCLM, which typically report primary technique efficacy above 85–90% and median overall survival ranging from 30 to 45 months [7,28]. The durable local control achieved here likely reflects careful patient selection, relatively small tumor size (median 19 mm), and contemporary ablation techniques using both radiofrequency and microwave energy. The low complication rate further supports ablation as a safe option in this population, particularly when repeat local treatment may be required over the course of metastatic disease.

The differential outcomes observed by molecular subtype are biologically plausible. HER2-positive patients demonstrated significantly improved overall survival, consistent with the profound survival gains seen with modern HER2-targeted therapies [20,29,30]. In contrast, triple-negative disease was associated with worse local tumor control and shorter chemotherapy-free survival, reflecting its more aggressive biology and limited targeted treatment options. While the small number of triple-negative cases precludes definitive conclusions, these findings highlight the importance of integrating tumor biology into multidisciplinary decision-making when considering liver-directed therapies. Given the small subgroups, the limited number of events, and the consequently wide confidence intervals, the molecular-subtype and treatment-intent associations reported here are exploratory and hypothesis-generating and require confirmation in larger cohorts.

An additional salient aspect of this study is the distinction between oligoprogressive and oligopersistent disease states. Patients treated for oligopersistence experienced significantly longer progression-free survival compared with those treated for oligoprogression, suggesting that disease biology and treatment timing may be as important as lesion count alone. This observation aligns with emerging conceptual frameworks for oligometastatic disease, which emphasize dynamic disease behavior rather than static numerical definitions [22,23], and with recent cohort data showing that metastasis-directed thermal ablation in visceral oligoprogressive or oligopersistent metastatic breast cancer can prolong post-ablation PFS while maintaining systemic therapy [31]. However, consistent with data from trials such as CURB, the benefit of local therapy in breast cancer—particularly in the oligoprogressive setting—appears heterogeneous and remains incompletely defined [21].

Several limitations merit consideration. This was a retrospective, single-center study with a modest sample size and inherent selection bias, limiting generalizability. The long inclusion period spans major advances in systemic therapy, which may have influenced outcomes independently of local treatment. Systemic regimens were heterogeneous and not analyzed in detail, and there was no non-ablated comparator group. As such, causality between ablation and prolonged systemic therapy benefit cannot be established. Furthermore, the study encompasses substantial evolution in ablation technology because modality choice correlated with both era and lesion characteristics; comparisons between RFA and MWA are confounded and cannot be interpreted causally. Nevertheless, the consistency of local control and the alignment of therapy-based endpoints with contemporary breast oncology priorities strengthen the clinical relevance of these findings.

## 5. Conclusions

Overall, these data support percutaneous thermal ablation as a valuable component of multidisciplinary care for selected patients with BCLM. When integrated with modern systemic therapy, ablation may provide durable local control while preserving systemic treatment options and delaying chemotherapy. In the absence of a comparator group, these observations are descriptive and cannot establish that ablation caused the observed benefit; the intervals may partly reflect favorable patient selection and concurrent systemic therapy. Prospective, multi-center studies incorporating standardized definitions of oligometastatic disease and patient-centered endpoints will be essential to further define the optimal role of ablation alongside surgery and stereotactic radiotherapy in metastatic breast cancer.

## Figures and Tables

**Table 1:** Baseline patient characteristics.

Patient Characteristics		Value (%)
Age–year		
	Median	53.5
	Interquartile range	44–66.5
Female sex		46 (100)
Tumor size–mm		
	Median	19
	Interquartile range	13–27
No. of tumors at first ablation		
	1	23 (50)
	2	14 (30)
	≥3	9 (20)
Extrahepatic disease		
	Yes	20 (43)
	No	26 (57)
Molecular subtype		
	HR+	26 (57)
	HER2+	13 (28)
	TN	7 (15)
BRCA Status		
	Positive	7 (15)
	Negative	35 (76)
	Unknown	4 (9)
Systemic treatment at the time of ablation		
	Targeted therapy	35 (76)
	Chemotherapy	5 (11)
	No treatment	6 (13)

## Data Availability

The data are available from the corresponding author on reasonable request, subject to institutional governance and patient confidentiality restrictions.
